# Primary and Secondary Caregivers of People with Dementia (PwD): Differential Patterns and Implications for Psychological Support

**DOI:** 10.3390/healthcare10061102

**Published:** 2022-06-14

**Authors:** Arantxa Gorostiaga, Igone Etxeberria, Karmele Salaberria, Iñigo Kortabitarte

**Affiliations:** 1 Department of Clinical and Health Psychology and Research Methods, Faculty of Psychology, University of the Basque Country UPV/EHU, Avda. Tolosa, 70, 20018 Donostia, Spain; igone.etxeberria@ehu.eus (I.E.); mcarmen.salaberria@ehu.eus (K.S.); 2 OKencasa, Parque Tecnológico de Miramón-Edificio Tándem, Paseo de Miramón 170, Planta 3, Módulo 18, 20009 Donostia, Spain; ik@okencasa.com

**Keywords:** primary and secondary caregivers, dementia, psychological health

## Abstract

Very little attention has been paid to identifying the differential characteristics of primary and secondary dementia caregivers. The aims of this study were: to determine whether differences exist between primary and secondary caregivers of people with dementia (PwD) and to explore the profile of primary and secondary caregivers reporting symptoms of anxiety and/or depression. The participants were 146 caregivers of PwD, 73 primary caregivers and 73 secondary caregivers. The results revealed different patterns for each type of caregiver. Primary caregivers showed a more negative profile in terms of poorer self-rated health and higher levels of anxiety and depression: 61.6% of primary and 42.5% of secondary caregivers reported symptoms of anxiety, and 24.7% and 11% reported depression, respectively. The frequency of problem behavior, subjective burden, health, and the comorbidity between anxiety and depression were associated with depression and anxiety among primary caregivers, whereas gender (being a woman), subjective burden, health, and the comorbidity between anxiety and depression were associated among secondary caregivers. These findings may help to guide professionals in targeting psychological support programs and customizing the strategies and skills that need to be provided in accordance with the type of caregiver in question: primary or secondary. The practical implications of the findings are discussed.

## 1. Introduction

The vast majority of studies carried out over the last three decades have focused on the effects that caring for a person with dementia (PwD) has on primary caregivers, who are usually the spouse or partner of the care recipient and live with them, providing round-the-clock care [1]. However, in addition to the primary caregiver, there are often one or more additional caregivers, usually the children of the care recipient, who offer secondary support and are known as secondary caregivers [2]. The tasks they perform include the following: (a) support in the basic activities of daily living (e.g., eating, dressing, and washing); (b) support in the instrumental activities of daily living (e.g., shopping and managing finances and medication, etc.); and (c) emotional support [3,4]. In a study comparing primary and secondary caregivers, which aimed to determine whether differences existed in their experience of care, Gaugler et al. [5] observed that primary caregivers were older, had a lower education level, and were, in general, the spouses of the care recipients. In contrast, secondary caregivers tended to have another job (paid employment) and mostly cared for older dementia patients.

Some authors had suggested that caregivers of PwD have unique psychosocial needs compared to other caregivers [6]. Precisely, the negative consequences of care for primary caregivers have also been widely studied from the perspective of stress models of caregiving [7,8], and evidence of their existence has been reported by many different authors. Some group these negative consequences into three dimensions: physical, psychological, and social [9]. In regard to health, caregivers generally have poorer health [10], a greater risk of developing cardiovascular disease [11], higher mortality [12], a higher accelerated aging of the immune system that has negative consequences for their health in comparison with non-caregiving counterparts [13], and more chronic pain, fatigue [14], and sleep problems [10].

In relation to psychological health, it has been reported that being a caregiver is linked to suffering higher distress [15] and more anxiety [10] than non-caregivers. It has also been found that as many as one third of caregivers suffer from clinical depression [16]. Recently published meta-analyses have reported an adjusted pooled prevalence of depression of 31.24% [17] and a pooled estimate of anxiety prevalence of 32.1% [18].

Finally, in terms of social consequences, previous research has reported that caregivers are more isolated, since they have less time for social, leisure, and spare time activities [10,19].

Little is known, however, about how caring for a PwD affects secondary caregivers. One of the few studies that has addressed this topic concluded that once certain other variables (such as frequency and amount of care provided and years spent caring for the care recipient) are controlled for, no significant differences exist between primary and secondary caregivers in terms of subjective health [5]. Nevertheless, other authors have found that primary caregivers have higher levels of caregiver burden and depression, take more psychotropic medication, have lower levels of physical health and life satisfaction, and participate fewer in social activities than their secondary counterparts [20]. Subsequent studies have confirmed that primary caregivers spend more time on care tasks and feel more overburdened than secondary caregivers [1], report more financial stress, and their health is worse in comparison with secondary caregivers; however, emotional stress and physical strain are similar in both types of caregivers [21]. Moreover, in a recent study authors found that, after controlling for some variables (caregiver’s demographics, caregiving arrangements, social support, sense of coherence, and neuropsychiatric symptoms of the person with dementia), there were differences between primary and secondary caregivers in subjective burden, while there were no differences in the positive aspects of caregiving [22].

Thus, although research has begun to recognize that caregiving is often a shared experience, little is known about those individuals who provide additional support (secondary caregivers). A more comprehensive understanding of the significant others involved in the support of PwD is crucial, since focusing exclusively on primary caregivers may provide an incomplete picture of the caregiver context. The aims of the present study were therefore: (a) to determine whether differences exist between primary and secondary caregivers of PwD in terms of their sociodemographic characteristics and care contexts (type of task performed, cohabitation, years of care provision, and frequency and intensity of care), as well as in terms of the consequences of care (self-rated health, anxiety, and depression); and (b) to explore the profiles of primary and secondary caregivers reporting symptoms of anxiety and/or depression.

## 2. Materials and Methods

### 2.1. Participants

The participants were 146 caregivers of PwD, of which 73 were primary caregivers and 73 secondary caregivers of the same person. The primary caregivers fulfilled the following inclusion criteria: (a) they were the main caregiver of the PwD; (b) they were over 18 years of age; and (c) they had provided at least 6 months of care. Likewise, exclusion criteria were: (a) having a contractual relationship for care provision with the elderly PwD or with his/her family; (b) having a disease that made participation in the study difficult (advanced cancer, cognitive impairment, etc.).

The relatives of the primary caregivers who also provided care to the PwD and were willing to participate were considered secondary caregivers. It was the caregivers themselves who classified their caregiving role as either primary or secondary [5]. Secondary caregivers supported the primary caregiver in their care tasks, but did not see themselves as the main caregivers of the PwD. In all other aspects, however, they complied with the same inclusion and exclusion criteria as primary caregivers.

### 2.2. Measures

All the measures were collected by online (tablet or smartphone) questionnaires.

#### 2.2.1. Sociodemographic Characteristics and Care Context

These variables were evaluated using a questionnaire prepared ad hoc for the study. The questionnaire included three sections:

- *Sociodemographic variables pertaining to the caregiver*: Age, gender (man, woman), marital status (single, widowed, divorced, married, in a domestic partnership), children in their care (yes, no, no answer), kinship (spouse, partner, son/daughter, sibling, other relative), education level (no qualifications, primary or secondary qualifications, further education, vocational training, university degree). Example of an item: “Do you have children in your care?”
- *Care context*: Type of caregiver (primary, secondary), type of task (care organization, direct care), cohabitation (yes, no), years of care provision (less than 2, 3–6, over 7), frequency of care (every day, 3–4 days a week, 1–2 days a week, 1–2 days a month), daily hours of care provision (>12, 6–12, <5). Example of an item: “How many hours do you spend each day caring for him/her?”
- *Care recipient*: Age. Example of an item: “How old is the person you care for?”

#### 2.2.2. Care Recipient Data

*Basic activities of daily living* (BADL) were measured using the Spanish version of the Barthel Index (BI) [23], which measures the extent to which a person can function independently and has mobility in BADL (i.e., feeding, bathing, grooming, dressing, bowel control, bladder control, toilet use, chair transfers, ambulation, and stair climbing). This instrument yields a score between 0 and 100, with lower scores indicating increased disability. Example of an item: “Feeding. 10 = independent (He/she is able to use cutlery, cut food, use salt, spread butter, etc.); 5 = needs help (He/she needs help for any of the activities mentioned); 0 = unable (He/she needs to be fed)”. The α index of the instrument in the present sample was 0.93.

*Independent living skills* were assessed using the Spanish version of the Lawton Instrumental Activities of Daily Living Scale [24], which measures eight domains of function, including areas such as shopping, cooking, and managing finances. It yields a summary score of between 0 (low function) and 8 (high function). Example of an item: “Indicate his/her ability to perform the following activities: Shopping. 1. Takes care of all shopping needs independently. 2. Shops independently for small purchases. 3. Needs to be accompanied on any shopping trip. 4. Completely unable to shop.” The α index of the instrument in the present sample was 0.69.

*Problem behavior* was measured using the Memory and Behavior Problems Checklist (MBCL-A) [25], translated by Izal and Montorio [26]. This inventory comprises 30 items that measure the frequency of each problem and the subjective burden that generates in the caregiver. Frequency was evaluated on a Likert-type scale with response options ranging from 0 (the problem in question never occurs) to 4 (the problem occurs daily) and burden with response options ranging from 0 (the problem does not stress me at all) to 4 (the problem stresses me completely). In this study, only the first 17 items, which refer to problem behaviors, were used. Example of an item: “He/she engages in activities that may be dangerous to him/her or to other people”. In the present sample, the α indexes were 0.78 and 0.88 for frequency and subjective burden, respectively.

#### 2.2.3. Caregiver Data

*Self-rated health* was determined through the following item: “In general, would you say that your health is very good, good, fair, poor or very poor?”. This item has been confirmed as a valid measure of the subjective perception of health in national and international studies [27,28,29]. For this study, the variable was recategorized, grouping fair, poor, and very poor in the same category.

*Anxiety and depression* were measured using the Spanish version of the Hospital Anxiety and Depression Scale [30,31]. The scale comprises 14 items and was designed to evaluate anxiety and depression in non-psychiatric outpatient hospital services. Seven items were related to anxiety and seven were associated with depression; all were scored on a response scale ranging from 0 (never) to 3 (almost all day). The authors recommended that a score of over 8 on any individual scale be regarded as a doubtful case and a score of over 11 as a case in both subscales. Item examples: “I get a sort of frightened feeling as if something awful is about to happen: 3. Very definitely and quite badly, 2. Yes, but not too badly, 1. A little, but it doesn’t worry me, 0. Not at all” (anxiety); “I have lost interest in my appearance: 3. Definitely, 2. I don’t take as much care as I should, 1. I may not take quite as much care, 0. I take just as much care as ever” (depression). In the present sample, the α indexes were 0.85 and 0.82 for anxiety and depression, respectively.

### 2.3. Procedure

Participants were recruited over the course of 5 months, between September 2018 and February 2019. Informal caregivers of PwD were recruited via informative letters and flyers through key institutions (San Sebastián City Council, basic social services, BetiOn (tele-assistance service run by the Basque Government), health centers, pharmacies in the city of San Sebastian, and the Gipuzkoa Association of Relatives and Friends of People with Alzheimer’s and other dementias (AFAGI)). Both the letters and the flyers provided a contact telephone number linked to a call center in which calls were received from families interested in participating. The person at the call center took the family’s call, answered any questions they may have had, and, if they agreed to participate, collected the necessary information to check whether they were eligible for inclusion in accordance with the inclusion/exclusion criteria. Subsequently, the project team analyzed each case and, if they were deemed eligible, the family (primary and secondary caregivers) was included in the study. All participants completed and signed an informed consent document. The data gathered were stored in a safe environment with strict encryption protocols. All data protection and confidentiality protocols were fully respected at the strictest level in accordance with that stipulated in the new European Data Protection Regulation (GDPR—General Data Protection Regulation). The study was approved by the Basque Ethics Committee for Research with Medicines (CEIm-E-PI2018086).

The data were gathered through self-report measures completed online (on a smartphone or tablet) at the caregivers’ homes, with the support of students from the Social Work undergraduate degree course who had been specifically trained for this task. The students resolved any doubts linked to how to complete the questionnaires.

### 2.4. Statistical Analysis

Descriptive data are presented with means and percentages. Primary and secondary caregivers were compared using Pearson’s chi square (*χ*^2^), with Cramer’s *v* effect size index in the case of categorical variables, and Student’s *t* statistic for independent samples, with Cohen’s *d* effect size index in the case of quantitative variables. Primary and secondary caregivers with and without depression and anxiety symptoms, according to the scores recommended by authors, >8 doubtful or case versus <8 no case, were compared using the same statistical analysis.

## 3. Results

### 3.1. Differences among Primary and Secondary Caregivers in Sociodemographic Characteristics, Care Context, and Consequences of Care

The differences between the two types of caregivers in terms of sociodemographic, care context, health characteristics, and characteristics of the care recipient are shown in Table 1.

Regarding sociodemographic variables, statistically significant differences were observed in age with a large effect size, the distribution of gender and employment situation with moderate effect sizes, kinship with relatively strong effect size, and whether or not they had children in their care with a smaller effect size.

Regarding care context and care recipient characteristics, statistically significant differences were found in type of task, cohabitation, frequency of care, and daily hours of care provision (with effect sizes ranging between moderate and relatively large). No statistically significant differences were found in any of the variables regarding the care recipient, and the effect sizes were small, indicating that primary and secondary caregivers had similar perceptions of the functional capacity and problem behavior of the person in their care.

Finally, statistically significant differences were observed between primary and secondary caregivers in psychological variables (anxiety and depression) and self-rated health with moderate effect sizes (see Table 1). Over half (61.6%) of the primary caregivers reported symptoms of anxiety, as opposed to 42.5% of secondary caregivers, with these figures being 24.7% and 11% (respectively) in relation to symptoms of depression. In addition, 34.2% of primary caregivers had fair, poor, or very poor perceived health, as opposed to 19.2% of secondary caregivers.

### 3.2. Profile of Primary and Secondary Caregivers with Symptoms of Depression and/or Anxiety

In order to compare the profile of primary and secondary caregivers who reported symptoms of anxiety and/or depression (cases) with that of those who did not (non-cases), measured in accordance with the >8 cutoff point recommended by the authors of the Hospital Anxiety and Depression Scale [30], the following variables were analyzed: sociodemographic characteristics, care context, and consequences of care (Table 2, Table 3, Table 4 and Table 5).

#### 3.2.1. Primary Caregivers

##### Anxiety

Statistically significant differences were found between the two groups (cases/non-cases) in terms of the years of care provision, the basic activities of daily living (BADL), the frequency of problem behaviors, and caregiver burden. In relation to health, statistically significant differences were found in depression and perceived health. Although it did not reach the significance level, moderate effect size was found in education level and the frequency of care. Specifically, primary caregivers reporting symptoms of anxiety tended to care for a PwD with more frequent problem behaviors. This gives rise to a greater caregiver burden, which in turn has a negative effect on caregiver health, with this group reporting not only symptoms of anxiety, but also symptoms of depression and poorer self-rated health. Moreover, the caregivers without anxiety had been taking care provision over seven years and a more dependent PwD in terms of BADL.

##### Depression

The results revealed statistically significant differences in the burden generated by problem behaviors, anxiety, and perceived health. Although they did not reach the statistical significance level, moderate effect sizes were observed for daily hours of care and frequency of problem behaviors. The results revealed that, in comparison with their non-depressive counterparts, caregivers with symptoms of depression tended to perceive more problem behaviors, resulting in a greater subjective burden, more anxiety, and poorer self-perceived health. Moreover, the non-depressive caregivers tended to care less hours a day.

#### 3.2.2. Secondary Caregivers

##### Anxiety

Statistically significant differences were found in the variables gender, frequency of problem behaviors, caregiver burden, perceived health, and depression. Moreover, although they did not reach statistical significance, years of care provision was found to have a moderate effect size. Specifically, secondary caregivers reporting symptoms of anxiety were mainly women and perceived more frequent problem behaviors in the PwD, reporting that these behaviors resulted in a greater subjective burden and depression. In contrast, non-cases of anxiety were more often men and had been taking care provision less than two years. Finally, secondary caregivers who perceived their health as being “very good” were mainly non-cases, while those who perceived their health as “fair”, “poor”, and “very poor” were mainly those reporting symptoms of anxiety.

##### Depression

The results revealed statistically significant differences in gender, children in their care, anxiety scores above the cutoff mark, and perceived health. Although they did not reach the statistical significance level, the effect sizes of the variables, basic and instrumental ADL, and stress caused by problem behaviors were moderate or medium (Table 2, Table 3, Table 4 and Table 5). Specifically, secondary caregivers reporting symptoms of depression were women with children in their care, tended to care for PwD who were more dependent in the basic and instrumental ADL, and felt more stressed as a result of their problem behavior (medium effect size); they also reported comorbid symptoms of anxiety and perceived fair, poor, or very poor health.

## 4. Discussion

The aims of the present study were to explore the possible differences between primary and secondary caregivers of PwD in terms of their sociodemographic characteristics, care contexts, and consequences of care (perceived health, anxiety, and depression), as well as to explore the profile of primary and secondary caregivers reporting symptoms of depression and/or anxiety.

### 4.1. Differences between Primary and Secondary Caregivers

The results revealed that primary and secondary caregivers do indeed have different profiles. Primary caregivers were characterized by being older than their secondary counterparts, as well as by mainly being women and spouses of the PwD, not having children in their care, and being retired or unemployed. For their part, secondary caregivers tended to be younger than their primary counterparts, and this group also contained a higher proportion of men. A higher percentage of secondary caregivers also had children in their care and had another kinship relationship with the care recipient (i.e., sibling/other relatives).

These results are consistent with those reported by Barbosa et al. [32], who found similar characteristics among Portuguese primary and secondary caregivers. Differences were also observed in relation to cohabitation, the type of task performed, and the frequency and intensity of the care provided. More primary than secondary caregivers live with the care recipient, and primary caregivers perform more direct care tasks such as helping with the activities of daily living (washing, feeding, transfers, etc.). They also provide more frequent and intense care in comparison with secondary caregivers (on a daily basis and for over 12 h a day). For their part, secondary caregivers perform more care organization tasks (tasks linked to the management and organization of the care provided). In addition, a greater percentage of them do not live with the care recipient and provide care 1–2 days a week or a month for less than five hours a day. These results prompt a reflection on the characteristics of those who are currently providing informal care to PwD in Spain. Primary caregivers are mostly the spouses of PwD, whereas secondary caregivers are mostly siblings or other relatives. A recent study analyzing the evolution of the number of potential caregivers of older adults with disabilities in Spain over the past 20 years (1998–2018) found results similar to those reported here for primary caregivers. The study identified two patterns. The first reflected an intergenerational flow of care, with members of younger generations looking after older, dependent individuals (i.e., their parents). The second pattern reflected an intragenerational flow of care, with caregivers and dependent persons being of a similar age [33], suggesting that care is provided mainly by spouses. According to our results, when the PwD is around 80 years of age, as in our study, and providing they are married, their primary caregiver is most likely their spouse. There is also likely to be a secondary caregiver who provides support and who is more probable to be another relative of the care recipient. When the care recipient does not have a spouse, it is usually one of their children who take on the role of primary caregiver, with another son or daughter or another relative acting as the secondary caregiver (it is worth noting that, in our sample, no spouse acted as a secondary caregiver). Our results also revealed that the second pattern (intragenerational) is mainly female, while in the first (intergenerational) there is a greater presence of men. This seems to point to an incipient change in gender roles in the field of care, particularly in terms of secondary caregivers from younger generations, a change that indicates a greater awareness among men of the importance of care. Given that men have begun to engage in care tasks, it would be interesting to study the differences that may exist between the two genders. In studies carried out to date, and as indeed found in the present study, women reported a greater burden associated with problem behaviors and provide more hours of care. Women also seem to suffer from role conflict, which forces them into the role of caregiver, whereas men view their care tasks from the perspective of problem solving, being less emotionally involved, and having more recourse to professional help [34,35].

Considering that dementia lasts, on average, between five and seven years, these caregiver profiles reflect very different situations and needs. Older caregivers are likely to have more health problems that may be aggravated by the negative consequences of care. This is because, as stated earlier in this paper, older individuals are more likely to be primary caregivers, and are therefore more vulnerable in terms of health and well-being since, according to our results, this group tends to have poorer self-rated health and higher levels of depression and anxiety than secondary caregivers. These findings are consistent with those reported previously by other authors [20], who argued that primary caregivers have higher levels of depression and poorer physical health than secondary ones. 

Our study also highlights the fact that primary caregivers have higher anxiety levels, a finding similar to Medrano et al. [36]. The percentage of those suffering from anxiety in our sample was fairly high, a finding that is even more important given the age of primary caregivers. From a clinical perspective, since little attention has been paid to date to anxiety disorders among the general older population, they have not been properly treated, an oversight which, according to Kaddour and Kishita [18], may have negative implications for general health. Thus, anxiety levels can compromise a caregiver’s ability to maintain their role effectively, at least in 1 out every 4 primary caregivers.

### 4.2. The Profile of Secondary Caregivers

The second aim of this study was to explore the profile of primary and secondary caregivers reporting symptoms of anxiety and/or depression. The differences observed in our study between primary caregivers who reported symptoms of anxiety and/or depression and those who did not were consistent with those found in other studies. Care recipient factors such as severity of dementia, problem behaviors, and subjective burden were found to be important risk factors for symptoms of depression [37,38,39,40] and anxiety among caregivers [39,41]. Poor self-rated health was also found to be associated with symptoms of anxiety and depression among primary caregivers, a finding that is consistent with that reported by international studies [42,43]. Finally, the high level of comorbidity observed between anxiety and depression is a finding that has been confirmed in systematic reviews [42]. In our study, we also found differences in terms of years of care provision, educational level, and frequency of care associated with anxiety and frequency of care with depression.

Whereas the profile of primary caregivers has been widely studied in the scientific literature, that of secondary caregivers has largely been overlooked, despite the fact that some studies claim that the presence of such a supporting figure is vital to ensuring the good mental health of the person principally responsible for care provision [44]. According to our results, secondary caregivers tend to be younger and include a greater proportion of men. They have diverse kinship ties with the care recipient (relatives other than children or spouses), tend to manage the care rather than directly care for the PwD, do not usually live with them, and spend less time on care tasks, probably because they need to combine this activity with others such as working or looking after the children in their care. Secondary caregivers generally enjoy better general health than their primary counterparts and have lower levels of both anxiety and depression. Nevertheless, a more detailed analysis revealed that a certain percentage of secondary caregivers reported probable or clinical cases of anxiety (42.5%) or depression (11%). The results of our study indicate the existence of certain variables that are associated with both depressive symptoms and anxiety. These variables are gender (being a woman), stress caused by problem behaviors, health, and the comorbidity between anxiety and depression. Other variables were found to be associated with either depression or anxiety: having children in their care and high levels of dependence in the basic and instrumental ADL were associated with depressive symptoms; frequency of problem behaviors and years of care provision were associated with anxiety. These results showed similar risk factors in both primary and secondary caregivers, referring to the subjective burden, health, anxiety, and depression.

In general, and as our results indicate, female caregivers, both primary and secondary, are more likely to be affected than their male counterparts [45], especially in terms of mental health [46]. Previous studies have found a greater prevalence of anxious-depressive symptoms among women than among men [47]. Regarding the subjective burden generated by problem behaviors, although we found no previous research analyzing the association between this factor and mental health among secondary caregivers, some studies have reported a positive association between problem behaviors and burden among this caregiver group [1]. As stated earlier, subjective burden is a variable that predicts anxiety and depression among primary caregivers.

The comorbidity observed here between depression and anxiety is consistent with that reported by studies focusing on primary caregivers. Most primary caregivers who report depressive symptoms also report symptoms of anxiety [48,49], although the reverse is not true (i.e., primary caregivers who report symptoms of anxiety do not necessarily have depressive symptoms also) [50]. In our study, the prevalence of anxiety among secondary caregivers was higher than that of depression, suggesting that the results found among primary caregivers may be applied also to secondary ones. In comparison with their symptom-free counterparts, secondary caregivers who reported symptoms of anxiety were more likely to be women, to perceive more frequent problem behaviors and subjective burden, as well as to rate their health as “fair”, “poor” or “very poor”. In terms of kinship ties, none of the secondary caregivers in our study were the spouse of the PwD, and non-cases were more often men. Another variable associated specifically with anxiety was the existence of problem behaviors. Previous studies reported that disruptive behaviors are important risk factors for anxiety symptoms among caregivers [40,41]. Problem behaviors among PwD exacerbate existing anxiety and stress among caregivers, and sustained situations of anxiety can often lead to sleep disorders and depression [51].

In relation to depression, when we compared the secondary caregivers who reported depressive symptoms with those who did not, we found that the former group more often had children in their care and cared for PwD who were more dependent in the basic and instrumental ADL. Having to combine their role as a parent with that of a caregiver of a PwD may increase the caregivers’ subjective burden. Moreover, secondary caregivers generally tend to feel that they could do more, and this may lead to feelings of guilt and ambivalence, which have been associated with higher levels of depression [52]. Although it has only been studied to date with primary caregivers, the severity of dementia, or in other words, a higher level of dependence in the basic and instrumental ADL, has been found to be an important risk factor for depressive symptoms [38,53].

## 5. Conclusions

In conclusion, differences exist between primary and secondary caregivers in terms of sociodemographic variables, care context, and care consequences (perceived health and psychological symptoms). A detailed analysis of secondary caregivers revealed that a percentage of them have mental health problems, and these findings suggest that caregivers may have different needs regarding their psychological health. To date, intervention programs have mainly been targeted at primary caregivers, and have been designed principally to provide them with strategies and psychological skills to help them cope adaptively with their care function [54,55]. Moreover, most are carried out face-to-face and use a group format.

However, the differential caregiver profiles found in our study suggest that individuals may have different needs in accordance with the type of caregiver they are, and they may even require different intervention formats.

In primary caregivers, greater attention should be paid to existing symptoms of depression and anxiety, and they should be encouraged to engage in positive, gratifying activities in order to counteract the negative effects of care. The management of problem behaviors is also of vital importance, given its strong association with caregivers’ mental health. In sum, awareness should be raised among primary caregivers of the fact that the support received from secondary caregivers is vital to their mental health and to enabling them to sustain their care activities over long periods of time [44].

Moreover, interventions targeted at secondary caregivers should be centered around two core issues: raising awareness of the situation of the primary caregiver to ensure the provision of adequate support; and preventing future health problems, such as anxiety and depression. It is vital to view the support provided by secondary caregivers as complementary rather than supplementary, which is how, in the majority of cases, it is understood today. Online interventions, which are becoming very popular in many countries, may be a good alternative for younger caregivers who have a higher education level and less time available for attending face-to-face intervention programs.

Family interventions designed to improve communication, family cohesion, and flexibility may serve as preventive strategies, helping to improve the mental health of both primary and secondary caregivers [56].

In light of the results found in the present study, a series of recommendation may be established from the perspective of public health. Physicians caring for PwD should take the health of the caregiver into consideration also, particularly when the PwD has certain problem behaviors. Moreover, gender should also be a point to consider when offering interventions designed to cover the specific needs of men and women, and a care network should be established that includes both primary and secondary caregivers. It is also crucial to design and implement social-educational campaigns focusing on the positive aspects of caregiving and the need to engage in this activity, since the vast majority of the population will require care at some point in their life.

This study has a series of limitations, one of which is the cross-sectional nature of the research. Longitudinal studies are required to analyze the evolution of primary and secondary caregivers of PwD as the disease progresses, as well as the evolution of the care dynamics. Not only would this provide greater insight into the evolution of caregivers themselves, but it would also help determine how their needs change over the course of the care process. Another limitation of this study is that the sample was comprised exclusively of caregivers living in an urban environment. This should be taken into consideration when interpreting and generalizing the results, and future research should strive to recruit more diverse samples. Although care was observed to have a greater impact on the psychological well-being of primary caregivers, a fairly large percentage of secondary caregivers also reported poor mental health. Future research may therefore wish to conduct a more in-depth analysis of the variables that predict diminished well-being among secondary caregivers.

Despite these limitations, however, our study highlighted the existence of differential patterns among those caring for PwD, specifically between primary and secondary caregivers, with the former generally having poorer self-rated and psychological health than the latter. This has clinical implications, since current intervention programs targeted at caregivers do not distinguish between the type of caregiver in question. Given that the principal aim of public institutions is to enable dependent persons to remain in their homes and delay their institutionalization for as long as possible, they should strive to provide differential caregiver support and aid programs, depending on whether the beneficiary in question is a primary or a secondary caregiver.

## Figures and Tables

**Table 1:** Comparison between primary and secondary caregivers in sociodemographic, care context, health, and care recipient characteristics.

	Primary *n* = 73		Secondary *n* = 73		*t*	*p*	*d*
Characteristic	*M*	*SD*	*M*	*SD*			
*Sociodemographic*							
Age	61.89	11.97	50.83	8.93	6.22	0.001	1.05
	***n***	***%***	***n***	***%***	***χ* ^2^**	***p***	***v***
Gender					8.43	0.004	0.24
Man	19	26	36	49.3			
Woman	54	74	37	50.7			
Marital status					0.73	0.394	0.07
Single/widowed/divorced	24	35.8	28	43.1			
Married/in a domestic partnership	43	64.2	37	56.9			
Children in their care					4.46	0.035	0.18
Yes	13	19.1	23	35.4			
No/no answer	55	80.9	42	64.4			
Kinship					30.84	0.001	0.46
Spouse/partner	22	30.1	0	0			
Son/daughter	45	61.6	52	71.2			
Sibling/other relative	6	8.2	21	28.8			
Education level					2.61	0.271	0.14
No qualifications/primary or secondary qualifications	14	20.9	9	13.8			
Further education/vocational training	23	34.3	18	27.7			
University degree	30	44.8	38	58.5			
Employment situation					5.59	0.018	0.21
Employee/self-employed	38	55.9	49	75.4			
Other (retired, unemployed…)	30	44.1	16	24.6			
*Care context*							
Type of task					8.01	0.005	0.23
Care organization	24	32.9	41	56.2			
Direct care	49	67.1	32	43.8			
Cohabitation					29.29	0.001	0.45
Yes	45	61.6	13	17.8			
No	28	38.4	60	82.2			
Years of care provision					3.26	0.196	0.15
Less than 2	12	16.4	15	20.5			
3–6	34	46.6	41	56.2			
Over 7	27	37	17	23.3			
Frequency of care					30.29	0.001	0.46
Every day	61	83.6	30	41.1			
3–4 days a week	8	11	16	21.9			
1–2 days a week/a month	4	5.5	27	37			
Daily hours of care provision					35.14	0.001	0.49
>12	39	53.4	6	8.2			
6–12	11	15.1	19	26			
<5	23	31.5	48	65.8			
*Care recipient*	***M***	***SD***	***M***	***SD***	***t***	***p***	***d***
Age *M* (*SD*)	81.51 (11.24)						
BADL	46.58	34.80	49.45	34.76	0.50	0.618	0.08
IADL	1.84	1.45	1.54	1.68	1.06	0.293	0.19
MBCL frequency	27.63	10.37	27.03	9.28	0.37	0.712	0.06
MBCL burden	12.33	9.31	9.66	7.94	1.87	0.064	0.31
*Caregivers’ health*							
Total anxiety	8.64	4.32	7.19	3.9	2.86	0.005	0.47
Total depression	5.23	3.82	3.48	2.84	2.85	0.005	0.47
	***n***	***%***	***n***	***%***	***χ* ^2^**	***p***	***v***
Perceived health					7.41	0.025	0.23
Very good	5	6.8	14	19.2			
Good	43	58.9	45	61.6			
Fair/poor/very poor	25	34.2	14	19.2			

Notes: BADL = basic activities of daily living; IADL = instrumental activities of daily living; MBCL = Memory and Behavior Problems Checklist.

**Table 2:** Comparison of the sociodemographic characteristics of primary and secondary caregivers reporting symptoms of anxiety.

Characteristic	Primary *n* = 73							Secondary *n* = 73						
	Case *n* = 45		Non Case *n* = 28		*χ* ^2^	*p*	*v*	Case *n* = 31		Non Case *n* = 42		*χ* ^2^	*p*	*v*
	*n*	*%*	*n*	*%*				*n*	*%*	*n*	*%*			
Gender					0.02	0.875	0.02					8.86	0.003	0.35
Male	12	26.7	7	25				9	29	27	64.3			
Female	33	73.3	21	75				22	71	15	35.7			
Marital status					0.253	0.615	0.06					0.03	0.861	0.02
Single/widowed/divorced	16	38.1	8	32				10	71.7	18	43.9			
Married/in a domestic partnership	26	61.9	17	68				14	58.3	23	56.1			
Children in their care					1.56	0.211	0.15					1.82	0.178	0.17
Yes	10	23.8	3	11.5				11	45.8	12	29.3			
No/no answer	32	76.2	23	88.5				13	54.2	29	70.7			
Kinship					1.29	0.524	0.13					0.01	0.966	0.01
Spouse/partner	12	26.7	10	35.7				0	0	0	0			
Son/daughter	30	66.7	15	53.6				22	71	30	71.4			
Sibling/other relative	3	6.7	3	10.7				9	29	12	28.6			
Education level					2.72	0.256	0.20					0.97	0.615	0.12
No qualifications/primary or secondary qualifications	8	19	6	24				2	8.3	7	17.1			
Further education/vocational training	12	28.6	11	44				7	29.2	11	26.8			
University degree	22	52.4	8	32				15	62.5	23	56.1			
Employment situation					0.07	0.790	0.03					0.42	0.515	0.08
Employee/self-employed	24	57.1	14	53.8				17	70.8	32	78			
Other (retired, unemployed…)	18	42.9	12	46.2				7	29.2	9	22			

**Table 3:** Comparison of the care context, characteristics of the care recipient, and health status of primary and secondary caregivers reporting symptoms of anxiety.

Characteristic	Primary *n* = 73							Secondary *n* = 73						
	Case *n* = 45		Non Case *n* = 28		*χ* ^2^	*p*	*v*	Case *n* = 31		Non Case *n* = 42		*χ* ^2^	*p*	*v*
*Care context*	***n***	*%*	***n***	*%*				***n***	*%*	***n***	*%*			
Type of task					1.28	0.258	0.13					1.53	0.217	0.14
Care organization	17	37.8	7	25				20	64.5	21	50			
Direct care	28	62.2	21	75				11	35.5	21	50			
Cohabitation					1.84	0.175	0.16					0.09	0.767	0.03
Yes	25	55.6	20	71.4				6	19.4	7	16.7			
No	20	44.4	8	28.6				25	80.6	35	83.3			
Years of care provision														
Less than 2	10	22.2	2	7.1	6.28	0.043	0.29	3	9.7	12	28.6	3.92	0.141	0.23
3–6	23	51.2	11	39.3				20	64.5	21	50			
Over 7	12	26.7	15	53.6				8	25.8	9	21.4			
Frequency of care														
Every day	38	84.4	23	82.1	2.89	0.236	0.20	13	41.9	17	40.5	4.46	0.108	0.25
3–4 days a week	6	13.3	2	7.1				10	32.3	6	14.3			
1–2 days a week/a month	1	2.2	3	10.7				8	25.8	19	45.2			
Daily hours of care														
>12	23	51.1	16	57.1	0.70	0.706	0.10	1	3.2	5	11.9	1.86	0.395	0.16
6–12	8	17.8	3	10.7				8	25.8	11	26.2			
Less than 5	14	31.1	9	32.1				22	71	26	61.9			
*Care recipient*	***M***	***SD***	***M***	***SD***	***t***	***p***	***d***	***M***	***SD***	***M***	***SD***	***t***	***p***	***d***
BADL	54.55	32.80	33.75	34.66	−2.58	0.012	0.62	50.48	35.41	48.69	34.68	−0.22	0.829	0.05
IADL	1.89	1.52	1.71	1.27	−0.39	0.700	0.13	1.52	1.79	1.55	1.52	−0.07	0.946	0.02
MBCL frequency	30.15	9.39	23.57	10.75	−2.75	0.007	0.65	31.06	9.47	24.04	8.01	−3.4	0.001	0.80
MBCL burden	15.33	9.60	7.50	6.43	−3.81	0.001	0.96	14.03	9.23	6.43	4.81	−4.5	0.001	1.03
*Caregivers’ health*														
Total depression	6.64	3.79	2.96	2.60	−4.51	0.001	1.13	5.64	3.10	2.11	1.79	6.11	0.001	1.39
	***n***	***%***	***n***	***%***	***χ*** **^2^**	***p***	***v***	***n***	***%***	***n***	***%***	***χ*** **^2^**	***p***	***v***
Perceived health														
Very good	1	2.2	4	14.3	10.16	0.006	0.37	2	6.5	12	28.6	19.98	0.001	0.52
Good	23	51.1	20	71.4				16	51.6	29	69			
Fair/Poor/Very poor	21	46.7	4	14.3				13	41.9	1	2.4			

Notes: BADL = basic activities of daily living; IADL = instrumental activities of daily living; MBCL = Memory and Behavior Problems Checklist.

**Table 4:** Comparison of the sociodemographic characteristics of primary and secondary caregivers reporting symptoms of depression.

Characteristic	Primary *n* = 73							Secondary *n* = 73						
	Case *n* = 18		Non-Case *n* = 55		*χ* ^2^	*p*	*v*	Case *n* = 8		Non-Case *n* = 65		*χ* ^2^	*p*	*v*
	*n*	*%*	*n*	*%*				*n*	*%*	*n*	*%*			
Gender					0.04	0.845	0.02					4.87	0.027	0.26
Male	5	27.8	14	25.5				1	12.5	35	53.8			
Female	13	72.2	41	74.5				7	87.5	30	46.2			
Marital status					0.01	0.958	0.01					0.01	0.990	0.01
Single/widowed/divorced	6	35.3	18	36				3	42.9	25	43.1			
Married/in a domestic partnership	11	64.7	32	64				4	57.1	33	56.9			
Children in their care					0.28	0.593	0.06					4.46	0.035	0.26
Yes	4	23.5	9	17.6				5	71.4	18	31			
No/no answer	13	76.5	42	82.4				2	28.6	40	69			
Kinship					0.96	0.620	0.11					1.16	0.281	0.13
Spouse/partner	7	38.9	15	27.3										
Son/daughter	10	55.6	35	63.6				7	87.5	45	69.2			
Sibling/other relative	1	5.6	5	9.1				1	12.5	20	30.8			
Education level					0.84	0.656	0.11					1.31	0.518	0.14
No qualifications/primary or secondary qualifications	4	23.5	10	20				0	0	9	15.5			
Further education/vocational training	7	41.2	16	32				2	28.6	16	27.6			
University degree	6	35.3	24	48				5	71.4	33	56.9			
Employment situation					0.08	0.778	0.03					0.07	0.797	0.03
Employee/self-employed	9	52.9	29	56.9				5	71.4	44	75.9			
Other (retired, unemployed…)	8	47.1	22	43.1				2	28.6	14	24.1			

**Table 5:** Comparison of the care context, characteristics of the care recipient, and health status of primary and secondary caregivers reporting symptoms of depression.

Characteristic	Primary *n* = 73							Secondary *n* = 73						
	Case *n* = 18		Non-Case *n* = 55		*χ* ^2^	*p*	*v*	Case *n* = 8		Non-Case *n* = 65		*χ* ^2^	*p*	*v*
	*n*	*%*	*n*	*%*				*n*	*%*	*n*	*%*			
*Care context*														
Type of task					1.23	0.268	0.13					0.14	0.710	0.04
Care organization	4	22.2	20	36.4				4	50	37	56.9			
Direct care	14	77.8	35	63.6				4	50	28	43.1			
Cohabitation					1.13	0.288	0.12					0.32	0.573	0.07
Yes	13	72.2	32	58.2				2	25	11	16.9			
No	5	27.8	23	41.8				6	75	54	83.1			
Years of care provision					2.04	0.360	0.17					2.69	0.261	0.19
Less than 2	2	11.1	10	18.2				0	0	15	23.1			
3–6	11	61.1	23	41.8				5	62.5	36	55.4			
Over 7	5	27.8	22	40				3	37.5	14	21.5			
Frequency of care														
Every day	16	88.9	45	81.8	1.39	0.499	0.14	3	37.5	27	41.5	1.37	0.503	0.14
3–4 days a week	2	11.1	6	10.9				3	37.5	13	20			
1–2 days a week/a month	0	0	4	7.3				2	25	25	38.5			
Daily hours of care														
>12	11	61.6	28	50.9	5.98	0.050	0.29	1	12.5	5	7.7	0.95	0.622	0.11
6–12	5	27.8	6	10.9				1	12.5	18	27.7			
Less than 5	2	11.1	21	38.2				6	75	42	64.6			
	***M***	***SD***	***M***	***SD***	***t***	***p***	***d***	***M***	***SD***	***M***	***SD***	***t***	***p***	***d***
*Care recipient*														
BADL	45.28	32.56	47.00	35.79	0.18	0.857	0.05	28.75	40.59	52	33.44	1.81	0.074	0.63
IADL	1.69	1.18	1.89	1.53	0.43	0.668	0.15	0.86	1.21	1.61	1.71	1.13	0.264	0.51
MBCL frequency	31.50	9.34	26.36	10.45	−1.85	0.068	0.52	30.25	8.86	26.63	9.32	−1.04	0.301	0.40
MBCL burden	17.50	10.85	10.64	8.16	−2.85	0.006	0.72	13.62	6.67	9.61	7.93	−1.37	0.133	0.60
*Caregivers’ health*														
Total anxiety	12.11	3.25	7.50	3.70	−4.70	0.001	1.32	11.50	3.34	6.12	3.76	−5.3	0.001	1.51
	***n***	***%***	***n***	***%***	***χ*** **^2^**	***p***	***v***	***n***	***%***	***n***	***%***	***χ*** **^2^**	***p***	***v***
Perceived health					15.63	0.001	0.46					6.37	0.041	0.30
Very good	0	0	5	9.1				0	0	14	21.5			
Good	5	27.8	38	69.1				4	50	41	63.1			
Fair/Poor/Very poor	13	72.2	12	21.8				4	50	10	15.4			

Notes: BADL = basic activities of daily living; IADL = instrumental activities of daily living; MBCL = Memory and Behavior Problems Checklist.

## Data Availability

The data presented in this study are available upon request from the corresponding author.
